# Structural and Functional Reorganization of the Brain in Migraine Without Aura

**DOI:** 10.3389/fneur.2019.00442

**Published:** 2019-05-07

**Authors:** Sourena Soheili-Nezhad, Alireza Sedghi, Ferdinand Schweser, Amir Eslami Shahr Babaki, Neda Jahanshad, Paul M. Thompson, Christian F. Beckmann, Emma Sprooten, Mansoureh Toghae

**Affiliations:** ^1^Donders Institute for Brain, Cognition, and Behaviour, Radboud University Medical Centre, Nijmegen, Netherlands; ^2^Donders Centre for Cognitive Neuroimaging, Radboud University, Nijmegen, Netherlands; ^3^Medical Informatics Laboratory, Queen's University, Kingston, ON, Canada; ^4^Department of Neurology, Jacobs School of Medicine and Biomedical Sciences, Buffalo Neuroimaging Analysis Center, University at Buffalo, Buffalo, NY, United States; ^5^Center for Biomedical Imaging, Clinical and Translational Science Institute, University at Buffalo, Buffalo, NY, United States; ^6^Cardiology Department, Iran University of Medical Sciences, Tehran, Iran; ^7^Keck School of Medicine of USC, Imaging Genetics Center, USC Stevens Neuroimaging and Informatics Institute, University of Southern California, Marina del Rey, CA, United States; ^8^John Radcliffe Hospital, Oxford Centre for Functional MRI of the Brain, Oxford, United Kingdom; ^9^Headache Department, Iranian Center of Neurological Research, Neuroscience Research Institute, Tehran University of Medical Sciences, Tehran, Iran

**Keywords:** functional connectivity, tensor-based morphometry, default-mode network, visual cortex, independent component analysis

## Abstract

It remains unknown whether migraine headache has a progressive component in its pathophysiology. Quantitative MRI may provide valuable insight into abnormal changes in the migraine interictum and assist in identifying disrupted brain networks. We carried out a data-driven study of structural integrity and functional connectivity of the resting brain in migraine without aura. MRI scanning was performed in 36 patients suffering from episodic migraine without aura and 33 age-matched healthy subjects. Voxel-wise analysis of regional brain volume was performed by registration of the T1-weighted MRI scans into a common study brain template using the tensor-based morphometry (TBM) method. Changes in functional synchronicity of the brain networks were assessed using probabilistic independent component analysis (ICA). TBM revealed that migraine is associated with reduced volume of the medial prefrontal cortex (mPFC). Among 375 functional brain networks, resting-state connectivity was decreased between two components spanning the visual cortex, posterior insula, and parietal somatosensory cortex. Our study reveals structural and functional alterations of the brain in the migraine interictum that may stem from underlying disease risk factors and the “silent” aura phenomenon. Longitudinal studies will be needed to investigate whether interictal brain changes are progressive and associated with clinical disease trajectories.

## Introduction

The current state of clinical practice in migraine is focused on reducing disease morbidity and frequency of headache attacks. However, there is ongoing debate regarding the potential long-term impacts of migraine on brain integrity and various domains of cognitive function, raising the critical question of whether migraine is accompanied by some progressive neuropathology with irreversible outcomes (1, 2).

From a genomics perspective, migraine is a multifactorial disease with a highly polygenic background (3). However, there are a number of rare migraine-resembling disorders caused by disruption of single genes, such as CADASIL, MELAS, and subtypes of familial hemiplegic migraine; in these disorders, recurrent headache is accompanied by serious complications including arteriopathy, subcortical infarcts, and brain atrophy (4). This line of evidence may indicate that chronic headache and progressive neuropathology sometimes share some close genetic and biological underpinnings. Of note, migraine headache, as a disease with a significant heritable component (5) (44–52%), is also associated with an increased risk of stroke in patients with aura (6) and a higher prevalence of stroke-like white-matter lesions in areas supplied by the posterior circulation (7). Nevertheless, association of migraine without aura, the most prevalent subtype of migraine, with subclinical brain pathology remains elusive.

Quantitative neuroimaging has revealed that migraine is associated with a palette of neurobiological events in the course of ictal attacks as well as in the prodromal and postictal phases (8). Among these, persistent brain changes that extend to the interictal intervals may be more informative on a potentially progressive and irreversible component of migraine. In this regard, quantitative brain morphometry provides useful information at the mesoscale resolution of MRI and indirectly reflects the loss of neural circuits and synaptic structures in various diseases. Structural brain morphometry also reveals regional reinforcement of neural pathways, which sometimes results in an increase in regional brain volume or cortical thickness (9). A number of voxel-based morphometry studies suggest that migraine is associated with a reduced volume of the insula, anterior cingulate, frontal cortex, and parietal operculum (10–13). Thickening of cortical areas engaged in visual and somatosensory processing as well as increased volume of periaqueductal gray matter has also been observed in migraine patients (14–16). While the relevance of such volumetric brain changes to migraine pathophysiology remains unknown, pain circuit dysmodulation has been the prevailing explanation (16).

Positron-emission tomography and functional MRI (fMRI) have revealed marked hypoperfusion of the occipital cortex as a heralding event in the cortical spreading depression (CSD) phenomenon in migraine attacks (17). fMRI has also identified spatiotemporal progression of the CSD in the human brain (18). In addition to these changes, which are temporally coupled to headache attacks, more recently, functional connectivity among several brain networks has been investigated using resting-state fMRI in the headache-free states, showing that intrinsic synchronicity of a number of brain regions is altered in the absence of headache symptoms, including the frontoparietal, anterior cingulate, and visual cortex (19–22). Causal relevance of brain connectivity changes to headache is unknown, and these findings may highlight some persistent brain traits that predispose to migraine, or rather, reflect a gradually progressive component of chronic ictal attacks.

It is noteworthy that the majority of the fMRI studies of migraine have focused on assessment of hypothesis-based brain networks, such as the executive (19, 21), visual (22), limbic (20), periaqueductal gray (23), salience (24), and default-mode networks (DMNs) (25–27). Probably due to disease heterogeneity and methodological differences, fMRI studies have provided conflicting results so far, with some contradictory reports of a lack of connectivity changes in migraine (27, 28). To our knowledge, a hypothesis-free whole-brain approach has not yet been used to evaluate changes in synchronicity of all resting brain networks in patients with migraine without aura.

In this study, we aimed to perform an exploratory assessment of brain networks in the interictal intervals of patients with migraine without aura. Using probabilistic ICA analysis, we decomposed a total of 375 intrinsic brain networks and investigated their altered connectivity in the resting state. We further assessed volumetric brain differences at voxel resolution using tensor-based morphometry (TBM). Our results show that migraine without aura is associated with abnormal changes in the functional connectivity of the visual cortex, parietal somatosensory cortex, and DMN, circuits that have been previously implicated in the pathophysiology of CSD and chronic pain. The observed interictal changes may stem from migraine risk factors or reflect a brain response to recurrent headache episodes.

## Materials and Methods

### Participants

This study was carried out in accordance with the recommendations and approval by the research ethics committee of the Tehran University of Medical Sciences, no. 91-01-54-17491-55005, with written informed consent from all subjects in accordance with the Declaration of Helsinki. Patients were enrolled from a headache clinic in Tehran (2012–2015) after providing written informed consent, including 36 right-handed female patients with migraine without aura with no history of other chronic diseases, substance abuse, or medication overuse headache. The diagnosis of migraine without aura was made by the senior neurologist investigator (MT) according to the ICHD-3 beta criteria. Mean age (±standard deviation) of the patient group was 36.6 ± 8.8 years (range: 20–54 years). Subjects did not receive any prophylaxis treatment for at least 6 months before enrolment in this study. Acute therapy medications used by the patient group are provided in Table 1. Twenty-six patients (72%) reported a positive family history of migraine in their immediate relatives. An age-matched control group of right-handed healthy female subjects (*n* = 33), who did not report a history of chronic medical condition or migraine in their immediate relatives, was also enrolled. The level of education was not different between the healthy and migraine groups. Clinical characteristics of the study population are provided in Table 2.

**Table 1:** Migraine acute therapy medications used by the study population.

**Medication**	**Subjects**
Sumatriptan or rizatriptan	9
Ergotamine	2
Ibuprofen	18
Acetaminophen	14
Naproxen	4
Indomethacin	3
Diclofenac	2
Celecoxib	2
Mefenamic acid	1
Aspirin	1
Alternative/herbal	2

*Twenty two patients (61%) reported consumption of more than one analgesic agent*.

**Table 2:** Clinical characteristics of the study population.

	**Migraine**	**Healthy**
Subject count (female)	36 (36)	33 (33)
Age^*^ (years)	36.6 ± 8.8 (range: 21–54)	36.4 ± 9.4 (range: 20–54)
Headache frequency (episodes/month)	5.4 ± 5.6	–
Pain intensity (1–10)	8.2 ± 1.6	–
Disease duration (years)	12.1 ± 8.6	–
Family history of chronic headache	26 subjects (72%)	–

* *Values reflect average ± standard deviation*.

### Structural MRI Image Acquisition

Subjects underwent an MRI session using a 3.0-Tesla scanner (Trio Tim, Siemens, Erlangen, Germany) and a 12-channel receive-only head coil. Patients had been in a headache-free state at least 24 h before the time of the scan. Several imaging modalities were collected, including structural T1-weighted MRI and resting-state fMRI. A fiducial marker (vitamin E capsule) was attached to the right temple to ensure correctness of the left/right axis in the volumes.

A 6-min three-dimensional magnetization-prepared rapid acquisition gradient echo (MPRAGE) sequence was used to record brain structure at the isotropic resolution of 1 mm with the following parameters: repetition time (TR): 2,530 ms, echo time (TE): 3.5 ms, inversion time (TI): 1,100 ms, flip angle: 7°, and GRAPPA with an acceleration factor of 2.

### Structural MRI Image Preprocessing and Tensor-Based Morphometry

**Figure 1:**
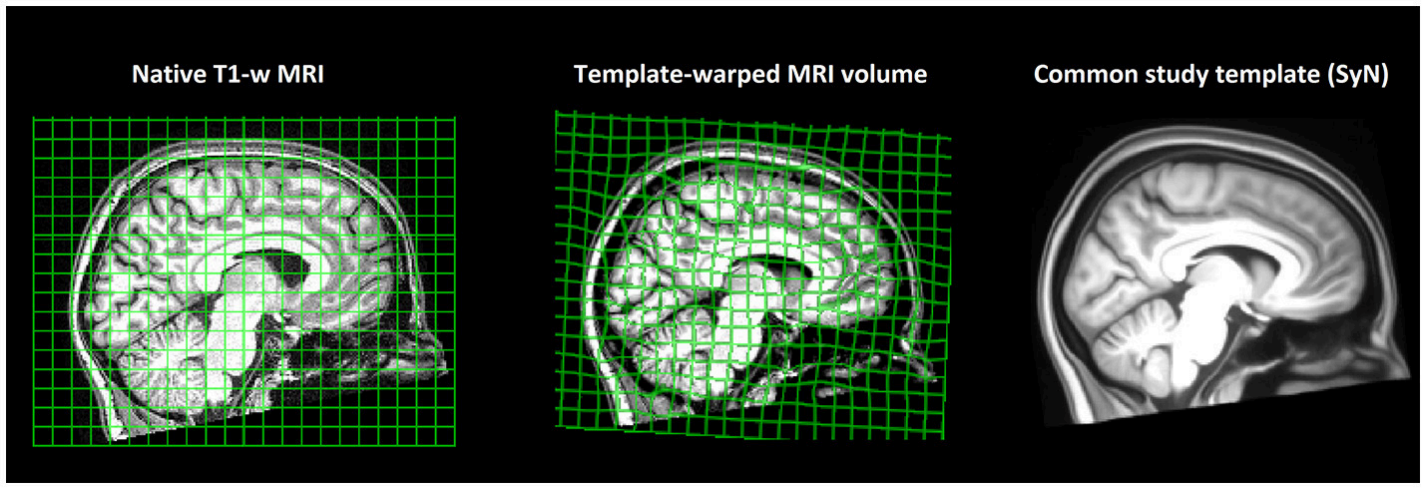
Study brain template. Subjects' T1-weighted MRI volumes (*n* = 69) were non-linearly registered to a common space, and a minimum-deformation template that represented an “average” study brain geometry was constructed. Non-linear warps transforming each subject's native brain geometry to the common study template were calculated, converted to Jacobian determinant fields, and compared across study groups by tensor-based morphometry (TBM).

MRI volumes were corrected for magnetic field bias using the N4 algorithm (29). Thereafter, all 69 T1-weighted volumes of the study population were recursively registered to construct a minimum-deformation template (Figure 1) by the diffeomorphic registration method *SyN*, part of the ANTS tool kit (30). The non-linear deformation field of each subject's brain was converted to Jacobian determinant maps encoding regional tissue shrinkage or expansion in relation to the average template (31). Thereafter, voxel-wise morphometry maps were compared between the migraine and healthy groups by the non-parametric linear model *Randomize* using 10,000 permutations (32), controlling for subject age. Voxel-wise statistics were corrected for multiple comparisons *via* threshold-free cluster enhancement (33).

### fMRI Image Acquisition and Denoising

Resting-state fMRI volumes were collected at the spatial resolution of 3.4 × 3.4 × 3.0 mm^3^ (matrix 64 × 64 × 40), 20% interslice gap, and an interleaved slice acquisition scheme. A total of 250 functional volumes were acquired with a repetition time (TR) of 2,570 ms, echo time (TE) of 33 ms, flip angle of 80°, and scan duration of 11 min. Three pairs of spin-echo echo-planar imaging volumes were also collected with opposing phase-encoding directions (A → P/P → A) for correcting image distortion using FSL *topup* (34). Subjects were asked to keep their eyes open and not to fall asleep or think about anything in particular. The effect of subject movement was corrected by linear alignment of volumes using *mcflirt*, and slice-timing correction was then applied using FSL *slicetimer*. The first six fMRI volumes were omitted to insure stabilization of the longitudinal magnetization, and functional volumes were then high-pass filtered (σ = 50 s) to eliminate low-frequency drifts of the signal. The preprocessed fMRI data were subsequently decomposed into independent spatial components by the *melodic* ICA algorithm to separate BOLD signals of neural origin from noise components (35). An automated denoising model was trained by manual labeling of components in 20 subjects (10 migraine patients and 10 healthy participants), and the trained model was used to autoclassify noise components of the rest of the study population (36). Time courses of noise components as well as 24 head motion regressors were then partialled out from the data using the non-aggressive approach (37). Finally, functional volumes were brought into the common brain template space by concatenating the subject's *fMRI*→*T1* linear transformation matrix with the *T1*→*template SyN* deformation field (30).

### Independent Component Analysis Decomposition of Intrinsic Brain Networks

The registered fMRI images of the whole study population were decomposed into spatially independent sources by FSL *melodic*, which linearly separated the data into a preset number of components. The independent component analysis (ICA) spatial maps were then regressed against each subject's denoised functional data to obtain component time courses (38). MELODIC was run four times, decomposing the brain's functional activity into 25, 50, 100, and 200 networks providing different levels of detail. Due to the large memory footprint of the input matrix (202,947 voxels × 16,592 frames), the –*migp* option was used in group ICA (39).

### Statistical Analysis of Functional Connectivity

To evaluate the strength of functional coupling between brain networks, Pearson's correlation coefficient of each of the two component time courses was calculated and transformed to the Gaussian distribution by Fisher's r-to-z transformation, yielding the node-to-node *network edge* strength values. Subject-wise edge strengths were then compared between the migraine and healthy populations while controlling for the confounding effect of subject age. Regression *p*-values were corrected for multiple comparisons across all studied edges by performing 5,000 random permutations.

## Results

### Structural MRI

**Figure 2:**
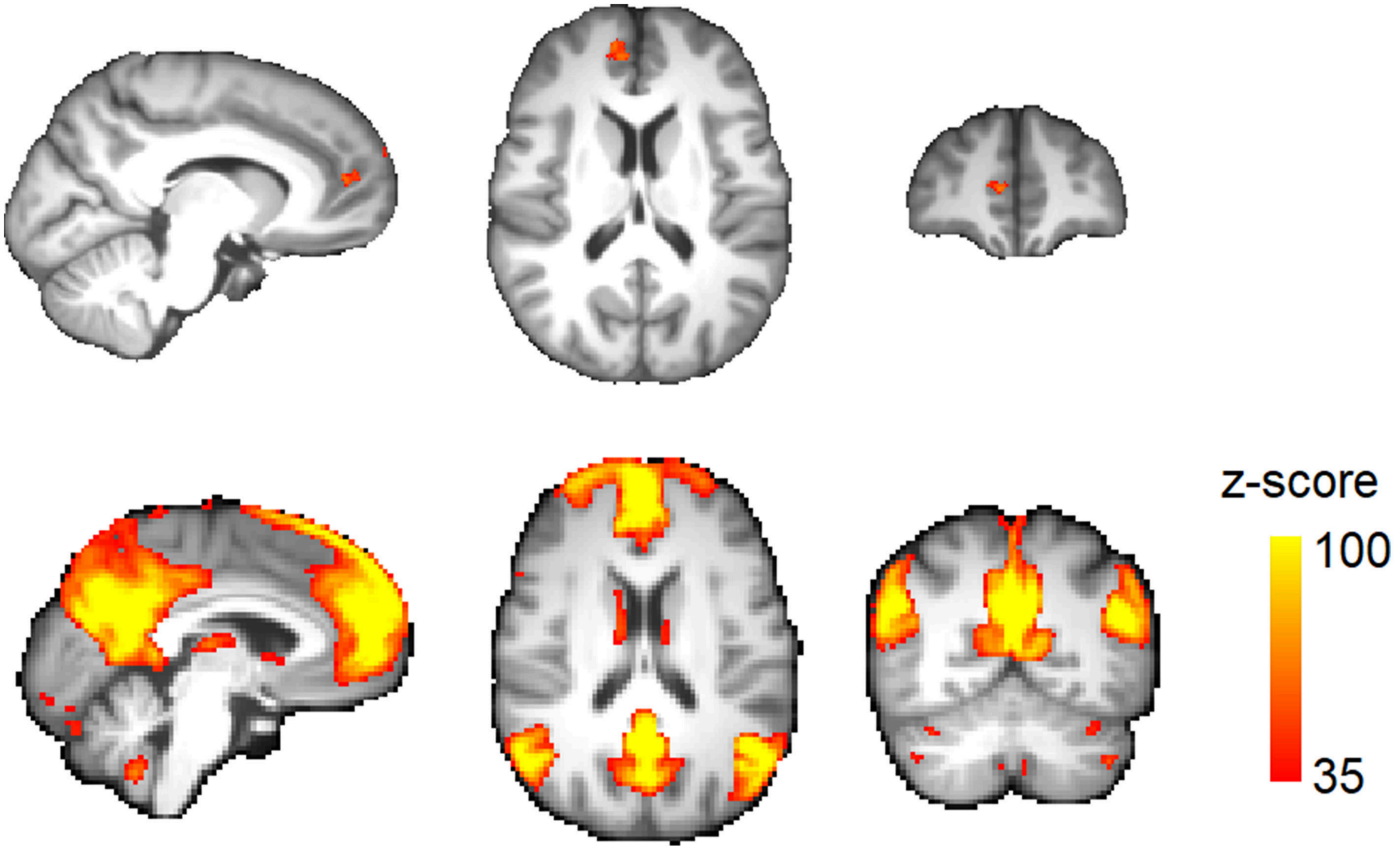
Regional brain volume loss in migraine. **(Top)** Voxels passing correction for multiple comparisons across the brain (TFCE) at corrected *p* < 0.05. **(Bottom)** A seed region was placed at the volume-loss region. Resting activity of this region was correlated with the default-mode network in the study population. The connectivity map is thresholded at *z*-score >35.

Total brain volume was not significantly different between migraine patients (1,217 ± 90 ml) and healthy subjects (1,202 ± 74 ml; *p* = 0.48). TBM analysis demonstrated a significantly lower brain volume in the right medial prefrontal cortex (mPFC) in migraine patients (Figure 2 and Table 3). No area of increased brain volume was observed in the patient group. By placing a seed region-of-interest in the mPFC volume-loss area, we observed its strong functional connectivity with the rest of the DMN in the study population (Figure 2).

**Table 3:** Standard MNI coordinates of brain volume deficits in migraine without aura.

**#cluster**	**Cluster vol. mm^**3**^ (TFCE)**	**Top p**	***X***	***Y***	***Z***
mPFC	17,164 (265)	2.4 × 10^−5^	8	51	8
Lt. middle frontal gyrus	12,836 (0)	2.4 × 10^−5^	−34	18	28
Rt. middle frontal gyrus	3,193 (0)	2.4 × 10^−5^	40	29	27

*MNI, Montreal Neurological Institute; TFCE, threshold-free cluster enhancement; mPFC, medial prefrontal cortex*.

### Resting-State fMRI

**Figure 3:**
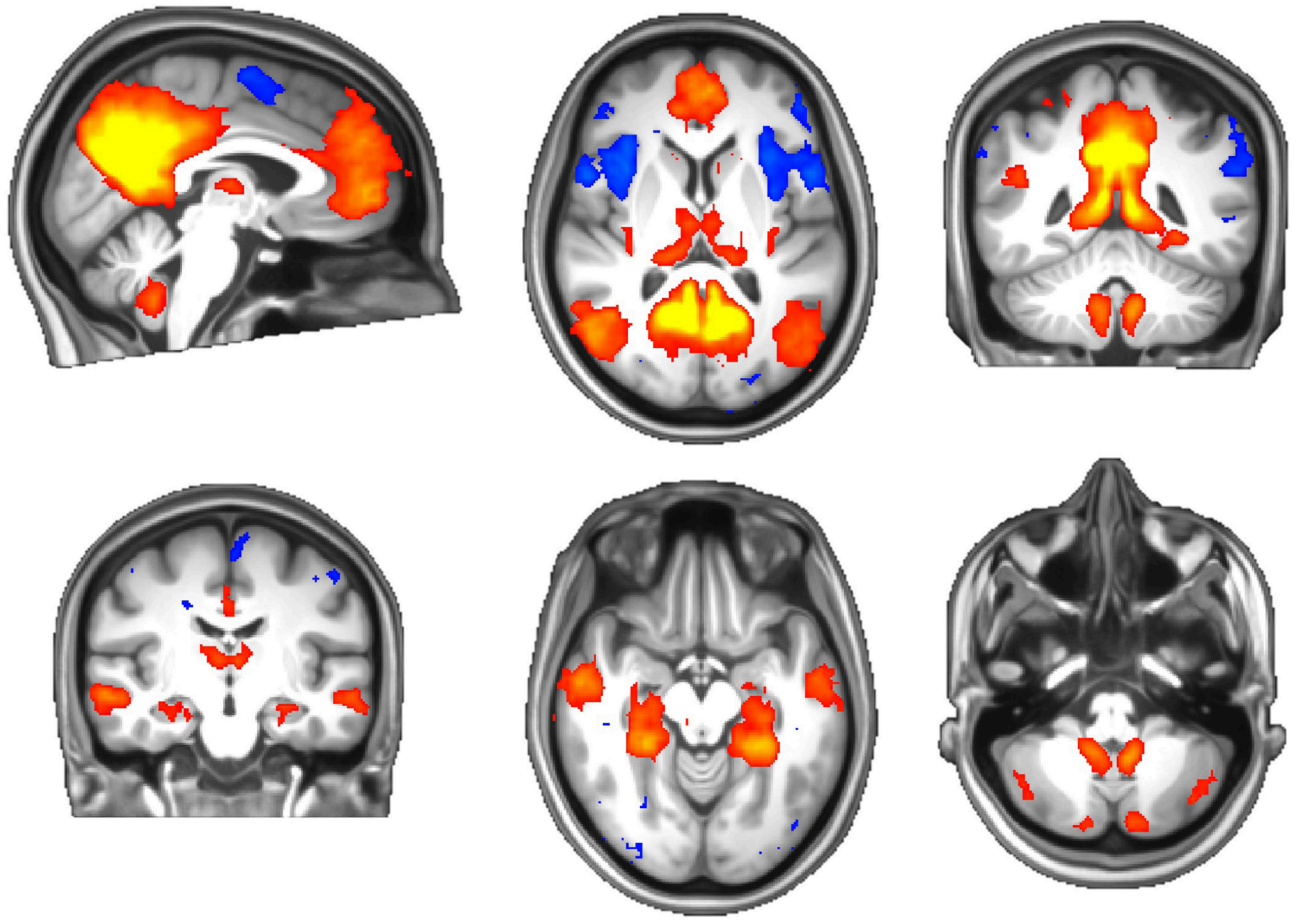
The default-mode network of the study population. Red-yellow: activation (correlation), blue: inhibition (anticorrelation).

There were no systematic differences in head movement parameters between the migraine and healthy groups. One subject's functional data were incompletely acquired due to an operator issue and omitted from the analysis. ICA revealed well-known resting brain networks in the study population, including the DMN (Figure 3).

**Figure 4:**
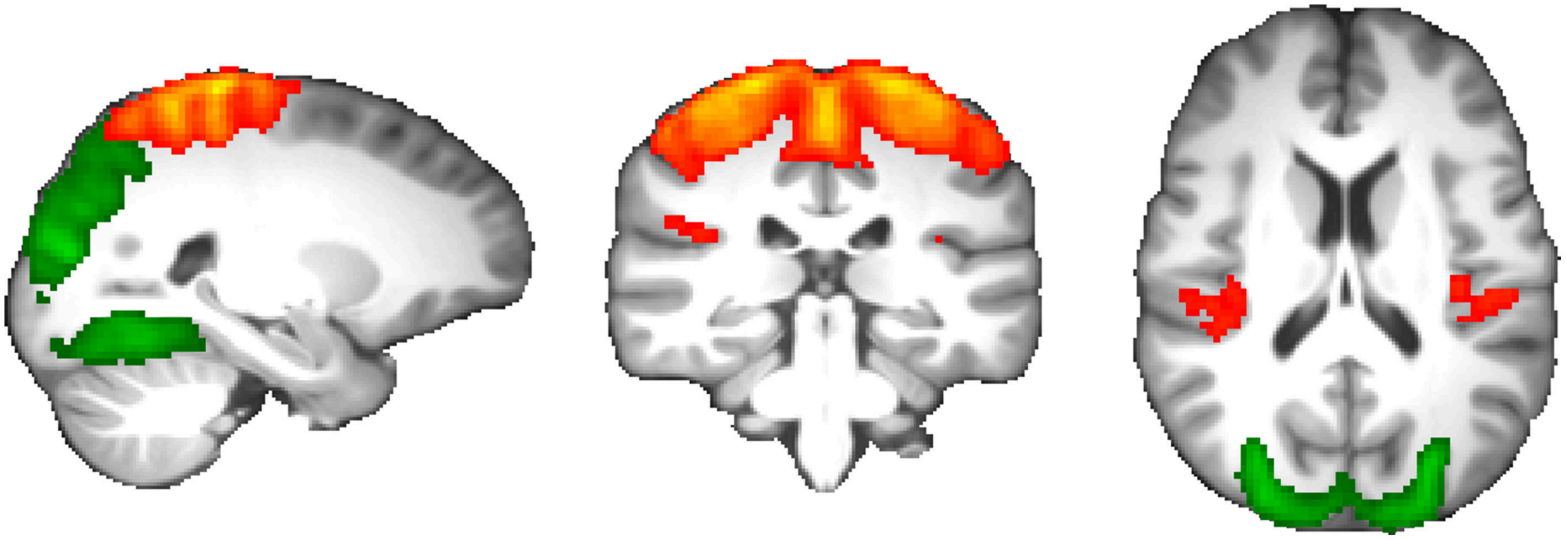
Resting-state functional connectivity changes in migraine without aura. Two components demonstrated significantly reduced functional coupling at *p* = 0.002, including the occipital cortex (green) and parietal-posterior insular cortex (red-yellow).

Comparing all network edge strengths between migraine patients and healthy subjects revealed statistically significant reduction of functional connectivity between two components spanning the occipital, postcentral, and precentral gyri; frontoparietal operculum; and posterior insular cortex that passed multiple comparisons correction (*p* = 6.4 × 10^−5^; permuted *p* = 0.002; Figure 4).

**Figure 5:**
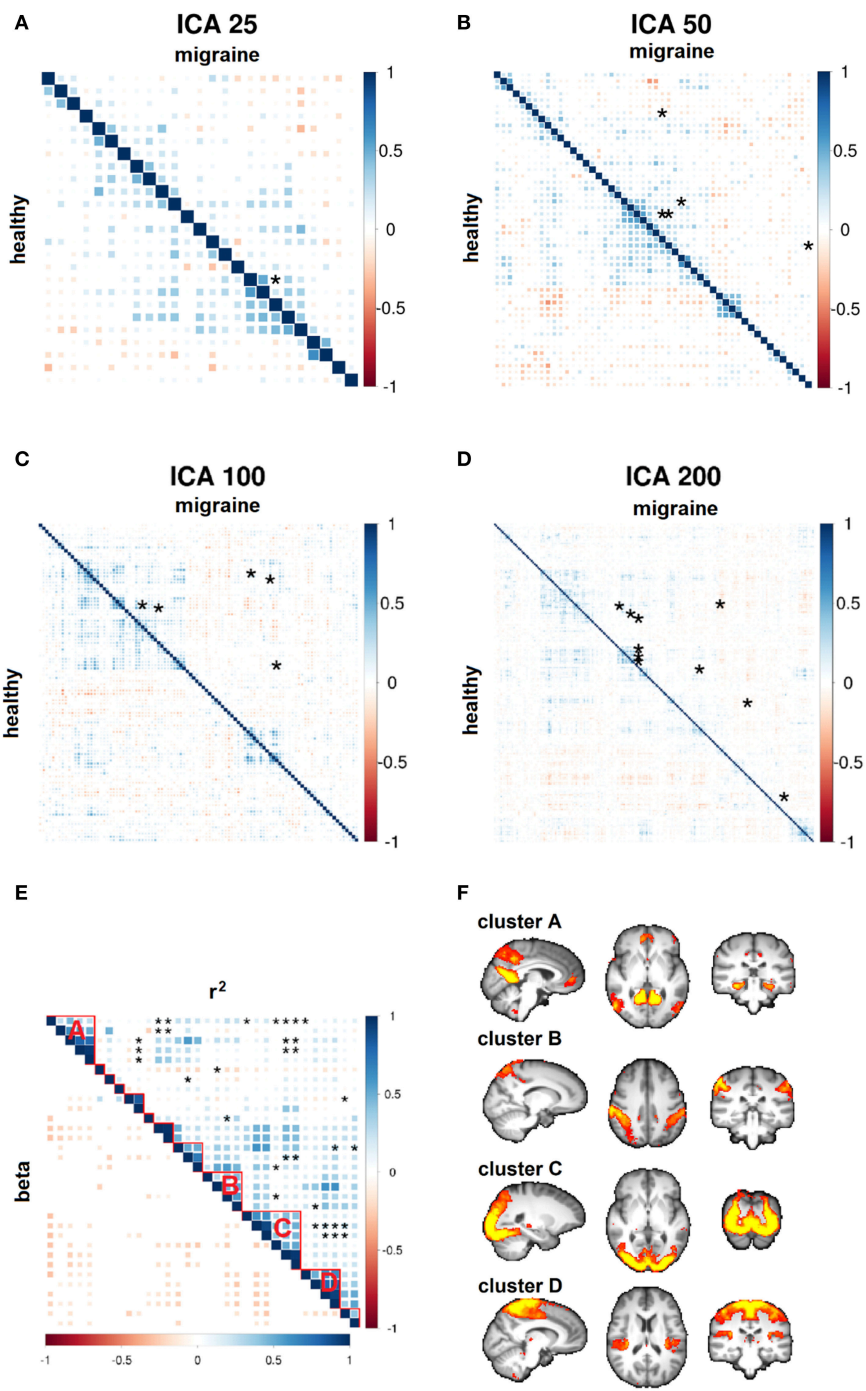
Whole-brain functional connectivity differences. **(A–D)** Node-to-node correlation matrices of independent component analysis (ICA) time course are shown at various dimensions. Network edges with suggestive connectivity differences in the migraine vs. healthy group (*p* < 10^−4^) are marked by asterisks. These disease-related edges are pooled in a single cross-correlation matrix for the purpose of visualization (**E**, top triangle). Spatially localized clusters of connectivity disruption are observed in migraine patients **(F)**. The sign of parameter estimates in regression models demonstrates generalized reduction of connectivity strength in migraine (**E**, bottom triangle; red-yellow: negative; blue: positive).

Increasing ICA dimensionality verified disruption of the same resting brain networks in higher spatial details with a similar direction of effects, although *p*-values did not pass the stringent multiple comparisons correction of larger connectivity matrices (Figures 5A–D). Importantly, of the total of 228 suggestively modulated network edges at *p* < 0.05 (uncorrected for the total number of studied edges), 98.2% (*n* = 224) had an effect direction reflecting reduced functional connectivity in migraine patients (Figure 5E). By clustering all of these resting brain networks, four main clusters of connectivity reduction were observed in migraine (Figure 5F), including the DMN (cluster A); bilateral intraparietal sulci (cluster B); occipital visual network (cluster C); and superior frontoparietal, insular, opercular, and thalamic network (cluster D).

### Neuroimaging Traits and the Clinical Measures of Migraine

No significant correlation was observed between structural and fMRI changes and measures of migraine severity including disease duration, headache intensity, and frequency. Power analysis in terms of required sample sizes to observe a significant correlation was performed (Supplementary Material). Disease duration showed the largest effect size, and structural MRI demonstrated higher power in tracking effects of this clinical measure than fMRI. The minimum sample size for observing the strongest correlation with the power of 80% was equal to *n* = 109 subjects.

## Discussion

Activity of the resting brain is a rich source of information for understanding its function in health and malfunction in disease (40). Although the structural deficits of mPFC in migraine may be an isolated phenomenon, we speculate that this finding may be related to the observed functional dysconnectivity of the DMN in which the mPFC actively engages. Previous evidence reveals that mPFC synchronicity within the DMN is associated with pain rumination (41). Lower connectivity of the mPFC with the rest of the DMN has also been observed in other chronic pain disorders, including osteoarthritis and low back pain (42, 43). While heterogeneous, results of previous structural MRI studies indicate that decreased volume of the frontal cortex is the most consistent observation in migraine (10, 12, 15, 44–48), with one study reporting lower volume of mPFC in patients with migraine without aura, similar to our work (13). At the synaptic level, recurrent painful stimuli lead to inhibition of the pyramidal neurons in mPFC through a GABAergic mechanism (49), and the same pathway may underpin the plasticity of mPFC in chronic pain conditions.

We observed lower connectivity of the visual cortex with the default-mode, frontoparietal, and posterior insular networks as the main hub of functional dysconnectivity in migraine. The interictal changes we observed in migraine may stem from abnormal neurodevelopmental pathways, underpinning cortical hyperexcitability (50). The visual cortex is the main site for initiation of oligemia and CSD that herald an ictal attack (17). Evidence supports hyperexcitability of the visual cortex in the interictal state of patients with migraine with and without aura (51). Structural alterations have also been observed in the V3A visual cortex of patients with migraine with and without aura (14), and our work adds another line of evidence to this “silent” aura phenomenon in the patients without aura. However, caution must be taken in drawing conclusions, since observation of structural changes in the visual cortex seems to depend on methodology (52).

The DMN has been previously implicated in the perception and processing of painful stimuli (42, 53). Reduced connectivity of the DMN has been observed in migraine without aura (25, 54), and specifically, the 0.04–0.08 Hz frequency band of this network shows decreased activity (55). The posterior insula, which demonstrated decreased connectivity to the visual cortex in our work, is the most specific region for the perception of pain in humans and acts as the homolog of the primate nociception center (56, 57). It is noteworthy that structural changes were observed in the anterior component of the DMN in the mPFC region, while functional dysconnectivity was observed in its posterior connections to the visual cortex and the insular nociception center. More research is needed to investigate a potential role of the DMN as an integral part of the pain circuit in migraine patients. The DMN may transfer abnormal headache-triggering neural activities from the visual cortex to downstream areas engaged in perception and cognitive processing of pain.

## Limitations

Due to a cross-sectional design, making any causal inference is difficult based on our findings. We did not observe any significant correlation between the MRI changes and various measures of disease severity such as pain intensity, attack frequency, and disease duration. This lack of correlation has been previously encountered (21, 22) and may indicate the need for higher statistical power with larger sample sizes to obtain clinically relevant neuroimaging biomarkers for migraine. Our findings run contrary to two reports of increased (27) and unaltered (28) functional connectivity in migraine using a dual regression approach but are in line with the reduced brain connectivity in migraine observed by a node-to-node correlation approach, which is more similar to our method (26). Given the heterogeneity of findings, there is a need for studies with larger sample sizes.

## Conclusions

In summary, we report abnormalities in the interictal state of the migraineur brain as reflected in the reorganization of the visual, somatosensory, insular, and DMNs. Until larger studies with longitudinal designs are performed, the relevance of these neuroimaging changes to underlying disease mechanisms will remain elusive.

## Data Availability

The datasets for this study will not be made publicly available because the ethical terms of the study prohibit public sharing of identifying subject-level information. All of the group-level study data including the brain template and brain network maps are available by contacting the corresponding author.

## Ethics Statement

This study was carried out in accordance with the recommendations and approval by the Research Ethics Committee of Tehran University of Medical Sciences, No. 91-01-54-17491-55005 with written informed consent from all subjects in accordance with the Declaration of Helsinki.

## Author Contributions

SS-N, AS, ES, and MT conceived the study, collected and analyzed the data, and wrote the manuscript. FS edited the manuscript. AE collected the data. NJ, PT, and CB jointly supervised the work and revised the manuscript.

### Conflict of Interest Statement

The authors declare that the research was conducted in the absence of any commercial or financial relationships that could be construed as a potential conflict of interest.
