# Supplementary material for: Structural and Functional Reorganization of the Brain in Migraine Without Aura
Source: Front Neurol. 2019 May 7;10:442. doi: 10.3389/fneur.2019.00442 (PMC6515892; doi:10.3389/fneur.2019.00442)
Supplement: Supplementary file 1 [file Data_Sheet_1.docx]

Supplementary table 1. Sample size estimates

|  | ICA-25 | ICA-50 | ICA-100 | ICA-200 | mPFC vol. (r) |
| --- | --- | --- | --- | --- | --- |
| Migraine duration | 315 | 369 | 419 | 459 | 109 (0.20) |
| Attack frequency | 1139 | 458 | 584 | 605 | 829 (-0.01) |
| Headache intensity | 419 | 608 | 594 | 542 | 631 (0.01) |

Numbers indicate the median sample size needed to find a significant correlation at α<0.05 with a statistical power of 80% across resting-state functional network edges which were modulated by migraine at p<0.05. The same sample size estimates are also provided for structural MRI changes in mPFC volume parameter and its Pearson’s correlation (r) coefficient with the three clinical measures. mPFC: medial prefrontal cortex.

# Supplementary methods

The effect size of each clinical measure of interest (*c*) in predicting the neuroimaging trait was calculated by the Cohen’s $f^{2}$ formula:

$$f^{2}=\frac{R_{age.c}^{2}-R_{age}^{2}}{1-R_{age.c}^{2}}$$

where $\boldsymbol{R}_{\boldsymbol{age.c}}^{\boldsymbol{2}}$ is the proportion of neuroimaging trait variance explained by both subject age and the clinical measure of interest together, and $\boldsymbol{R}_{\boldsymbol{age}}^{\boldsymbol{2}}$ is the unique proportion of neuroimaging trait variance explained by subject age alone. The sample size needed to obtain a target statistical power of β=0.8 was calculated.
